# The rural mortality penalty in U.S. hospital patients with COVID-19

**DOI:** 10.21203/rs.3.rs-3467683/v1

**Published:** 2023-11-10

**Authors:** Jeffrey A. Thompson, Dinesh Pal Mudaranthakam, Lynn Chollet-Hinton

**Affiliations:** University of Kansas Medical Center; University of Kansas Medical Center; University of Kansas Medical Center

**Keywords:** COVID-19, pandemic, rural mortality penalty, disparities

## Abstract

**Background:**

The COVID-19 pandemic brought greater focus to the rural mortality penalty in the U.S., which describes the greater mortality rate in rural compared to urban areas. Although it is understood that issues such as access to care, age structure of the population, and differences in behavior are likely drivers of the rural mortality penalty, it is critical to try and understand these factors to enable more effective public health policy.

**Methods:**

We performed a cross-sectional analysis of a population of patients with COVID-19 who were admitted to hospitals in the United States between 3/1/2020 and 2/26/2023 to better understand factors leading to outcome disparities amongst groups that all had some level of access to hospital care, hypothesizing that deteriorated patient condition at admission likely explained some of the observed difference in mortality between rural and urban populations.

**Results:**

Our results supported our hypothesis, showing that the rural mortality penalty persists in this population and that by multiple measures, rural patients were likely to be admitted in worse condition, had worse overall health, and were older.

**Conclusions:**

Although the pandemic threw the rural mortality penalty into sharp relief, it is important to remember that it existed prior to the pandemic and will continue to exist until effective interventions are implemented. This study demonstrates the critical need to address the underlying factors that resulted in rural-dwelling patients being admitted to the hospital in worse condition than their urban-dwelling counterparts during the COVID-19 pandemic, which likely affected other healthcare outcomes as well.

## Background

In the early 20^th^ century, dense population in cities with poor sanitation led to a phenomenon known as the urban mortality penalty. However, improvements in sanitation and increased effectiveness and accessibility to healthcare eventually eliminated this disparity(1). Indeed, mortality rates declined throughout the United States in the 20^th^ century(2). However, in 2008, Cosby et al. identified a new trend, beginning in the 1980s, in which a new rural mortality penalty became evident(3), leading to a host of research on location-based health disparities. Since its initial discovery, the rural mortality penalty has only increased(4). Such population health disparities can have acute impacts during a public health crisis, such as the COVID-19 pandemic that began in 2019. Indeed, many have noted that rural populations in the United States suffered increased mortality due to COVID-19 compared to their urban counterparts(5–7). When it comes to healthcare, a common assumption with rural disparities is that access to care plays a major role(8, 9). Our focus is on the persistence of the rural mortality penalty even in those patients with access to, means for, and willingness to go to a hospital. Therefore, in this work, we studied mortality in a population of patients with COVID-19 following their admission to a hospital, using a large national dataset of approximately a million COVID-19 patients who were admitted to hospitals to determine its drivers.

When viewing rural dwelling as a social determinant of health, it is important to note that other social determinants are associated with excess mortality, such as education, poverty, race, and more(10). In fact, Black Americans experience larger mortality disparities, irrespective of location, than rural populations(11), particularly with respect to the COVID-19 pandemic. Also, it is worth noting that the emergence of the rural mortality penalty has not, for most population groups, been due to increased mortality rates. In many areas, it has been driven by smaller declines in mortality in rural compared to urban populations(3, 4, 12). Nevertheless, although COVID-19 has made the trend harder to analyze, there is evidence to suggest that mortality is increasing in younger and middle-aged adults for certain causes of death (causes related to drugs and alcohol, as well as suicide) and that the group has overall seen smaller declines in mortality due to cardiovascular disease(13).

Others have written about rural/urban mortality disparities from COVID-19. Grome et al. studied county-level mortality data in Tennessee throughout most of 2020 and found an incidence rate ratio of 1.13 for mortality in rural vs. urban counties after controlling for county-level demographics, access to care, and comorbidities(5). Interestingly, they found that the disparity was greater for COVID-19 than for influenza or pneumonia. Iyanda et al. found similar results for a nationwide county-level analysis with a mortality incident rate ratio between 1.24 and 1.26 depending on the county classification(7), which also adjusted for county-level demographic and other factors. However, these studies did not examine COVID-19 mortality in the context of patients who were admitted to a hospital.

Although several studies on hospital outcomes for COVID-19 patients have been done(14–18), most have not evaluated location-based risk. One large national study conducted by Anzalone et al. examined whether the rural mortality penalty is present in COVID-19 patients following hospital admission, using data on approximately one million patients obtained from the National COVID Cohort Collaborative (N3C) (19). A strength of the study is that it analyzed in-hospital risk at the individual level. Indeed, they found an increased risk of in-hospital mortality for rural patients, with an adjusted odds ratio of 1.36. However, the study did not examine patient condition at admission or vaccination status. Therefore, the study was limited in its ability to determine the drivers of the rural mortality penalty with respect to COVID-19 hospital patients.

Several studies have examined factors contributing to hospital outcomes for COVID-19 patients without considering rurality. Two studies found that the effects of race on COVID-19 mortality in hospital patients was largely mitigated after adjusting for admitting hospital(14, 17). A different study found that once admitted to hospitals in New York, Black patients were less likely to die(20). Thus, it appears race as a risk factor for COVID-19 mortality following hospital admission is largely related to where the patient is admitted. Other factors affecting risk of COVID-19 mortality noted by multiple studies were age(14, 17, 21), sex(17, 21), and vital signs at admission(14, 21).

It is important to remember that the rural mortality penalty existed prior to the COVID-19 pandemic. The dramatic increase in mortality due to COVID-19 brought these existing disparities in health outcomes into sharp focus. However, the underlying drivers will remain. Furthermore, rurality, race, and most other social determinants of health are typically not causes of health outcomes. Instead, issues such as access to care, differences in demographics, differences in behavior, lower rates of belief in the efficacy of public health intervention(22–24), and many other factors are the true drivers. Therefore, it is critical to gain understanding of these drivers so we can work to improve the health of the entire population. We hypothesized that the rural mortality penalty in patients hospitalized with COVID-19 is driven by deteriorated patient health in the rural population prior to admission, in combination with increased risk factors in the rural population overall.

## Methods

### Data

We performed our analysis using Epic’s Cosmos database using the 2/26/2023 snapshot. We used the deidentified version of the database provided with Cosmos Data Science. At the time of this snapshot, the database contained data from 196 organizations across the United States comprising data on 186,082,759 patients. We identified COVID-19 admissions using the COVID patient data mart provided by Cosmos. For this data mart, COVID-19 admissions were identified as follows:

- COVID diagnosis linked to an encounter for an admitted patient that occurred before discharge and within 42 days of admission.
- Respiratory diagnosis linked to an encounter for an admitted patient that occurred before discharge and within 42 days of admission and is COVID positive between 14 days before admission and the discharge date and discharge is 42 days or less from the positive date.
- Patient became COVID positive during an admission and discharge date is 42 days or less from COVID positive date.

The outcome was all cause mortality within 42 days of a COVID related admission. We collected the following demographic data: sex, race, residence in rural zip code by Rural Urban Commuting Area (RUCA), social vulnerability index of zip code, and age at admission. We also collected the following patient condition variables (closest measure to admission): oxygen saturation, respiration rate, pulse rate, mean arterial pressure, and admitted to ICU within first day. Additionally, we collected the following comorbidities data: congestive heart failure, high blood pressure, pneumonia, and cancer. Finally, we also collected data on whether a patient had at least one of any type of COVID-19 vaccination two weeks or more prior to admission. Presumably due to the urgent nature of most COVID-19 admissions, BMI was not available for most patients.

We included patients with a first COVID admission between 3/1/2020 and 2/26/2023. Patients from sources with less than 50 COVID-19 patients were excluded to ensure that the results were not biased by hospitals who did not admit many COVID patients (17 such sources which included 142 patients). The study population included 1,119,199 patients in the database who met the definition of having a COVID-19 admission.

However, many patients were missing information on vital signs. While we included all eligible patients regardless of missing data, there were 648,595 patients that had no missing vitals and only 165,534 patients with COVID vaccination records in the hospitals they were admitted to.

### Statistics

The main variable of interest for this analysis was rural zip code residence as defined by RUCA codes, maintained by the U.S. Department of Agriculture. Patients were categorized in Cosmos as simply rural (codes 7–10) or urban (codes 1–6) dwelling. Patient characteristics were individually tested for differences by rural or urban dwelling using a Wilcoxon rank sum test for continuous variables or χ^2^ a for categorical variables. The unadjusted difference in risk of mortality for urban/rural differences was tested by log rank test and visualized using a Kaplan-Meier survival curve. The adjusted difference in risk was assessed using a Cox proportional hazards model using complete case analysis. The proportional hazards assumption was assessed visually, using the scaled Schoenfeld residuals plotted against the log time, because hypothesis tests of non-proportional hazards are suspect with such a large sample size. The assumption appeared warranted for all variables.

## Results

Table 1 shows all the putative risk factors for COVID mortality we included in this study, with summary statistics broken down by whether the patient lived in a rural or urban designated zip code. Hypothesis tests were done for each variable to test if they differed by rurality. For most demographic, patient condition, and comorbidity variables, there was a statistically significant difference between rural and urban patients.

**Figure 1:**
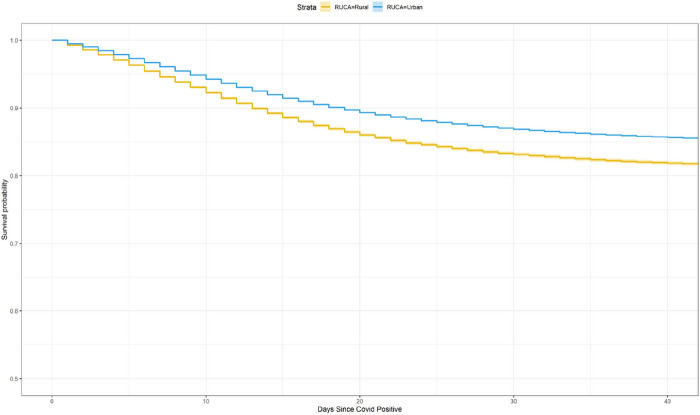
Kaplan Meier survival curves over the days since a patient was covid positive for rural vs. urban residing patients.

A Kaplan-Meier survival plot showing the unadjusted difference in survival between patients from rural or urban designated zip codes is shown in Fig. 1. It illustrates that patients from rural areas are at higher risk of mortality following admission for COVID-19. The unadjusted hazard ratio for this difference is 1.299 (95% CI: [1.276, 1.323]). The difference is significant at p < 0.001.

Univariable Cox proportional hazards models were used to estimate the individual effect on risk for each putative risk variable. The Hazard ratios for each of these variables are shown in Table S1, along with the concordance index. These results show that differences in age at diagnosis is highly concordant with increased COVID-19 mortality (C-index = 0.681). Each one-year increase in age is associated with about a 4% increase in the hazard ratio (p < 0.001). Meanwhile, rural residence alone is not very concordant with increased mortality (C-index = 0.510).

A multivariable Cox proportional hazards model was used to estimate the effect of rurality on risk adjusting for demographic differences, differences in patient condition at admission, and differences in comorbidities. The results are shown in Table 2. When controlling for other putative drivers of COVID-19 mortality, the effect of residence is attenuated. Rural residence has a hazard ratio of 1.139 (95% CI: [1.111, 1.168], p < 0.001) in this model. Congestive heart failure, pneumonia, and cancer comorbidities all contribute to risk, as do admission to the ICU, lowered blood oxygen, increased respiration, and increased pulse at admission. Low blood pressure was associated with higher risk; however, high blood pressure was associated with lower risk, compared to normal blood pressure. Social vulnerability was associated with increased risk, as was male sex. Black patients were at slightly lower risk compared to white patients after adjusting for other factors in this population. Finally, the hazards reduced over time, reflected in the hazard ratio of 0.999 for days since 3/1/2020.

**Figure 2:**
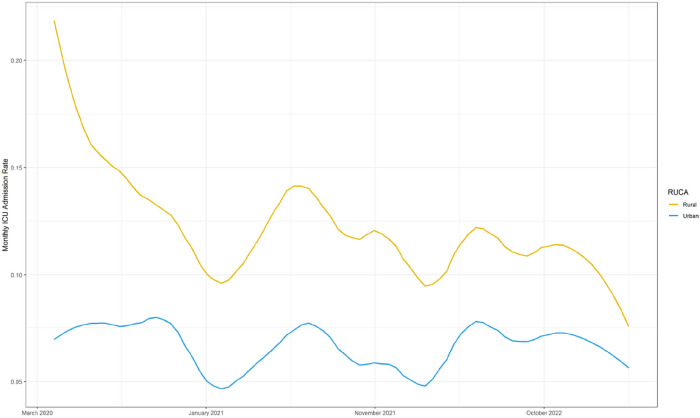
Monthly ICU admission rate in rural vs. urban residing patients.

In order to examine the difference in patient condition at admission to the hospital by area of residence, we plotted the monthly rate of ICU admission for rural and urban patients over the course of the pandemic (Fig. 2). Rural patients consistently have higher rates of admission to the ICU throughout the course of the pandemic.

We built an additional multivariable Cox proportional hazards model using the subset of patients that we knew had at least one COVID-19 vaccination prior to admission with COVID-19. In this model, the hazard ratio for urban residence became 1.109 (95% CI: [1.047, 1.174], p < 0.001), further reducing the hazard ratio of rural residence after accounting for vaccination.

## Discussion

The rural mortality penalty is thought to be associated with numerous factors, including reduced access to care, differences in behavior, education, environmental exposure, and other factors. Access to care issues can mean that people do not have access to hospital care, have delayed access to hospital care, or do not have access to the same quality hospital care. In this analysis, we examined a population of patients that all were admitted to a hospital and then adjusted for patient condition at admission in order to address differences in access to care between rural and urban populations, although we were unable to assess differences in quality of care. We also adjusted for differences in age and sex, which can skew differently in rural vs. urban populations. We adjusted for differences in race and social vulnerability in an attempt to account for other known drivers of disparities. However, race in this context should not be thought of as a biological variable; rather, it is a proxy variable that captures other access to care and risk factor differences resulting from structural inequalities.

After these adjustments, we did manage to reduce the effect of rural residence on COVID-19 mortality from a HR of 1.299 to 1.139, indicating that we had identified some but not all of the drivers of mortality disparities in these patients. Patient condition variables and age were the strongest contributors to this result, which likely point in part to access to care and differences in demographics as some of the main factors underlying the rural mortality penalty in COVID-19. However, patient condition at admission is also driven by differences in behavior, including diet, exercise, and vaccination.

Although hospitals do not have complete vaccination records for patients, some were available. Therefore, we built a multiple Cox proportional hazards model on the subset of 165,534 patients for whom we had a record of COVID-19 vaccination at least two weeks prior to hospital admission. Of those, 9,645 were from rural areas. Although the overall results were similar for this subset analysis, we did find that the increased hazards associated with rural residence shrank even more (compared to the adjusted model) to 1.109, suggesting that differences in vaccination may further contribute to the rural mortality penalty.

Limitations of this study include the lack of information about the individual hospitals included within our Cosmos dataset, which is an inevitable consequence of using a deidentified dataset, as well as the lack of complete vaccination records for patients, which would have enabled us to better assess the impact of vaccination on the rural mortality penalty in this context. Also, due the nature of COVID-19 admissions, we lacked information on BMI for most patients, although BMI is an important risk factor for COVID-19 mortality. Finally, the analysis was limited to hospitals that use the Epic electronic health record system, which does not tend to be used by smaller organizations. These limitations are counterbalanced to some degree by being able to perform the analysis in a nationwide dataset of patients admitted to hospital, with information on vitals, and ICU admission. Given the substantial cross-section of COVID-19 hospital admitted patients covered in this resource, these results are likely generalizable at least to relatively well-resourced hospitals.

Overall, we found that there are differences in COVID-19 mortality between rural and urban populations, even after limiting the analysis to patients admitted to hospitals and adjusting for patient condition, comorbidities, and demographics. However, the rural mortality penalty associated with COVID-19 hospital admission is largely the result of deterioration in condition before admission to hospital (which could be due to delayed access to care, more severe disease, lack of trust or belief in the importance of care, or other factors), age structure of the population, overall worse health of the rural population (as reflected in the greater number of comorbidities), and overall lower vaccination rates in the rural population. Likely, the residual effect of rurality is related to other comorbidities we did not account for, in addition to differences in behaviors such as diet, exercise, and vaccination.

## Conclusions

Despite the focus in this work on COVID-19 mortality in patients admitted to hospitals, we believe it adds to the body of evidence about the rural mortality penalty in general. Rural populations face reduced access to care, lower rates of belief in the efficacy of public health intervention, lower education levels, and lower rates of healthy behaviors such as exercise. The situation only stands to become worse, with continued closures of hospitals in rural areas. Therefore, in order to make a meaningful difference in reducing the rural mortality penalty, it will be important to meet rural populations where they are and work to create partnerships that build trust between communities and healthcare providers.

## Figures and Tables

**Table 1:** Characteristics of the study population

Variable	N	Rural, N = 77,807	Urban, N = 1,041,392	p-value
Comorbidities				
Congestive Heart Failure	1,119,199	8,130 (10.4%)	105,195 (10.1%)	0.002
High Blood Pressure	1,119,199	28,885 (37.1%)	386,886 (37.2%)	0.9
Pneumonia	1,119,199	4,610 (5.9%)	58,754 (5.6%)	<0.001
Cancer	1,119,199	8,059 (10.4%)	99,552 (9.6%)	<0.001
Patient Condition				
Mean Arterial Pressure	807,220			<0.001
(60,100]		39,309 (71.1%)	520,490 (69.2%)	
(0,60]		756 (1.4%)	8,772 (1.2%)	
(100,300]		15,191 (27.5%)	222,702 (29.6%)	
Unknown		22,551	289,428	
ICU Admission	1,119,199	8,732 (11.2%)	62,906 (6.0%)	<0.001
SpO2	911,943	95 (93, 97)	96 (93, 98)	<0.001
Unknown		14,137	193,119	
Respiration Rate	808,396	20 (18, 24)	20 (18, 23)	<0.001
Unknown		23,377	287,426	
Pulse Rate	906,257	86 (74, 99)	87 (75, 101)	<0.001
Unknown		16,632	196,310	
Demographics				
SVI	1,113,692	0.66 (0.46, 0.82)	0.53 (0.26, 0.79)	<0.001
Unknown		1,335	4,172	
Comorbidities				
Congestive Heart Failure	1,119,199	8,130 (10.4%)	105,195 (10.1%)	0.002
Sex	1,119,199			<0.001
Female		36,992 (47.5%)	519,128 (49.8%)	
Male		40,815 (52.5%)	522,264 (50.2%)	
Race	1,119,199			<0.001
White		65,276 (83.9%)	712,506 (68.4%)	
American Indian or Alaska Native		1,809 (2.3%)	9,350 (0.9%)	
Asian		252 (0.3%)	24,755 (2.4%)	
Black or African American		7,128 (9.2%)	214,787 (20.6%)	
Native Hawaiian or Other Pacific Islander		141 (0.2%)	3,962 (0.4%)	
Other		1,639 (2.1%)	49,573 (4.8%)	
Unspecified		1,562 (2.0%)	26,459 (2.5%)	
Age at Diagnosis	1,119,199	67 (54, 78)	65 (49, 77)	<0.001
Days from 3/1/2020 to Admission	1,119,199	573 (313, 710)	559 (309, 705)	<0.001

**Table 2 –:** Results of multivariable Cox regression. The concordance index for this model was 0.735.

Variable	Ln HR	HR	p-value
Residence			
Urban	0.000	1.000	-
Rural	0.130	1.139	< 0.001
Comorbidities			
Congestive Heart Failure	0.215	1.240	< 0.001
High Blood Pressure	−0.059	0.943	< 0.001
Pneumonia	0.148	1.160	< 0.001
Cancer	0.190	1.210	< 0.001
Patient Condition			
Mean Arterial Pressure			
(60,100]	0.000	1.000	-
(0,60]	0.718	2.051	< 0.001
(100,300]	−0.269	0.764	< 0.001
ICU Admission	0.826	2.284	< 0.001
SpO2	−0.030	0.971	< 0.001
Respiration Rate	0.028	1.029	< 0.001
Pulse Rate	0.008	1.008	< 0.001
Demographics			
SVI	0.261	1.299	< 0.001
Sex			
Female	0.000	1.000	-
Male	0.272	1.312	< 0.001
Race			
White	0.000	1.000	-
American Indian or Alaska Native	0.065	1.067	0.085
Asian	−0.032	0.968	0.196
Residence			
Urban	0.000	1.000	-
Black or African American	−0.092	0.912	< 0.001
Native Hawaiian or Other Pacific Islander	0.029	1.029	0.672
Other	0.041	1.042	< 0.001
Unspecified	0.425	1.530	< 0.001
Age at Diagnosis	0.044	1.045	< 0.001
Days from 3/1/2020 to Admission	−0.001	0.999	< 0.001

## Data Availability

The data that support the findings of this study are available in Epic’s Cosmos environment. This is an analytic environment from which data cannot be removed. Therefore, restrictions apply to the availability of these data, which were used under license for the current study, and so are not publicly available. Data are however available to healthcare institutions which use the Epic EHR and participate in Cosmos. Researchers at Epic institutions should contact their Epic technical support to get access to the data.

## Abbreviations

N3C: National COVID Cohort Collaborative
RUCA: Rural Urban Commuting Area
